# Preliminary results of a new endoscopic underlay cartilage tympanoplasty with lateral malleolar flap

**DOI:** 10.1007/s00405-025-09337-5

**Published:** 2025-03-28

**Authors:** Pinar Atabey, Burcu Vural Camalan, Hasan Demirel, Mehmet Beyhan Balur, Sumeyra Doluoglu

**Affiliations:** 1 Ankara Medical Park Hospital, Ankara, Turkey; 2 Department of Otorhinolaryngology Head and Neck Surgery, University of Health Sciences Ankara Etlik City Hospital, Ankara, Turkey; 3 Batman Dunya Hospital, Batman, Turkey; 4 Batman Yasam Hospital, Batman, Turkey; 5 Ankara Etlik City Hospital, Department of Otorhinolaryngology Head and Neck Surgery, University of Health Sciences Turkey, Varlık Mahallesi, Halil Sezai Erkut Caddesi, No:5, Yenimahalle, 06170 Ankara Turkey

**Keywords:** Cartilage tympanoplasty, Chronic otitis media, Endoscopic tympanoplasty, Lateral malleolar flap

## Abstract

**Background:**

A new endoscopic tympanoplasty technique for large and medium-sized central perforations via a transcanal approach, without tympanomeatal flap elevation compared the preliminary postoperative graft success and hearing outcomes with other techniques in the literature.

**Methods:**

The study involved 75 patients aged 12 to 44 who underwent tympanoplasty with the lateral malleolar flap technique from 2014 to 2017. Pre-operative otoscopy recorded perforation sizes, and pure tone averages were calculated at 500, 1000, 2000, and 4000 Hz. The procedure was performed under general anesthesia via a transcanal approach using tragal cartilage, without tympanomeatal flap elevation. A control otoscopy was conducted six months post-operation to assess reperforation and graft position. Pure tone averages were measured, and pre-operative and post-operative audiological values were compared.

**Results:**

Post-operative six-month graft success rate was 94.6%. Reperforation was observed in one patient (1.3%), while another patient (1.3%) exhibited lateralization, and two patients (2.6%) demonstrated medialization. The pre-operative mean hearing level was recorded at 33.3 ± 7.0 dB, accompanied by an Air-Bone Gap (ABG) of 24.0 ± 6.6 dB. Post-operative measurements indicated an improvement in these values, with the mean hearing level decreasing to 18.0 ± 4.8 dB and the ABG reducing to 16.3 ± 5.3 dB (*p* < 0.05).

**Conclusion:**

The endoscopic underlay cartilage tympanoplasty with lateral malleolar flap is a safe technique that avoids elevating the tympanomeatal flap. It offers shorter operation time, easier postoperative dressing, and high graft success rates similar to other tympanoplasty techniques.

## Introduction

Chronic otitis media with hearing loss is a severe problem that affects a large proportion of the population. Early diagnosis and treatment are crucial to resolving functional losses caused by this disease group. Chronic otitis media surgery primarily aims to keep the ear dry, close the perforation, prevent recurrent infection, and provide corrected hearing [1].

Tympanoplasty is a surgical procedure that involves cleaning the inflammation in the middle ear, repairing the tympanic membrane and auditory system in the middle ear [2]. From 1950 to 2000, surgical techniques improved with the development of microscopes. In the last 40 years, with the development of endoscopes, physicians’ experience in endoscopic surgery has increased.

Endoscopic tympanoplasty, introduced in the 1990s, is becoming more preferred by several surgeons due to its wider viewing angle and absence of postauricular incision. Endoscopic Type I Tympanoplasty (ETT) is currently accepted as an effective, minimally invasive procedure for repairing tympanic membrane perforations [3]. Different tissues and materials, such as skin grafts, fascia lata, temporal fascia, perichondrium, cartilage, and fat, have been used as grafts for tympanic membrane repair. Inlay cartilage tympanoplasty is a conservative method for repairing small and medium perforations. It involves using tragal and cartilage composite grafts with the perichondrium. This method is the most conservative compared to traditional tympanoplasty techniques [4].

In this study, a new endoscopic cartilage tympanoplasty approach was introduced to the literature, which could be applied in large and medium perforations through a transcanal approach by using membrane remnant in the lateral malleus as a superior-based flap with no external auditory canal elevation. The preliminary post-operative grafting success and hearing results of patients were shared.

## Methods

This study reviewed the medical records of 75 patients who underwent tympanoplasty with the endoscopic lateral malleolar flap technique at our clinic from 2014 to 2017. The study was approved by a Tertiary Health Center’s ethics committee (Date: 23.03.2021 approval no: 269) and informed consent were obtained from all participants of the study. Perforation sizes were measured during pre-operative otoscopy and categorized based on their ratio to total tympanum size: small (less than 1/3), moderate (1/3 to 2/3), and large (greater than 2/3). Perforation localization was assessed relative to the manubrium mallei. Anterior perforations did not involve the manubrium and were located anteriorly, while posterior and inferior perforations were classified accordingly. Central perforations involved the umbo and manubrium. Subtotal perforations had residual tympanic membrane tissue only in the pars flaccida.

All patients underwent tympanoplasty with a lateral malleolar flap, performed by the same surgeon under general anesthesia. The surgical approach involved a transcanal technique that incorporated tragal cartilage without the elevation of a tympanomeatal flap. Control otoscopy was conducted for all patients at the six-month post-operative mark to assess for reperforation, as well as graft medialization or lateralization. Both pre-operative and post-operative (sixth month) average Air-Bone Gap (ABG) and pure-tone audiometric results (at frequencies of 500, 1000, 2000, and 4000 Hz) were analyzed, along with the overall graft success rate. Demographic data for the patients were recorded. Exclusion criteria included individuals with age under 18 or over 70 years old, and patients with active purulent discharge and/or cholesteatoma and surgery requiring mastoidectomy or bone chain reconstruction.

### Surgical technique

**Fig. 1:**
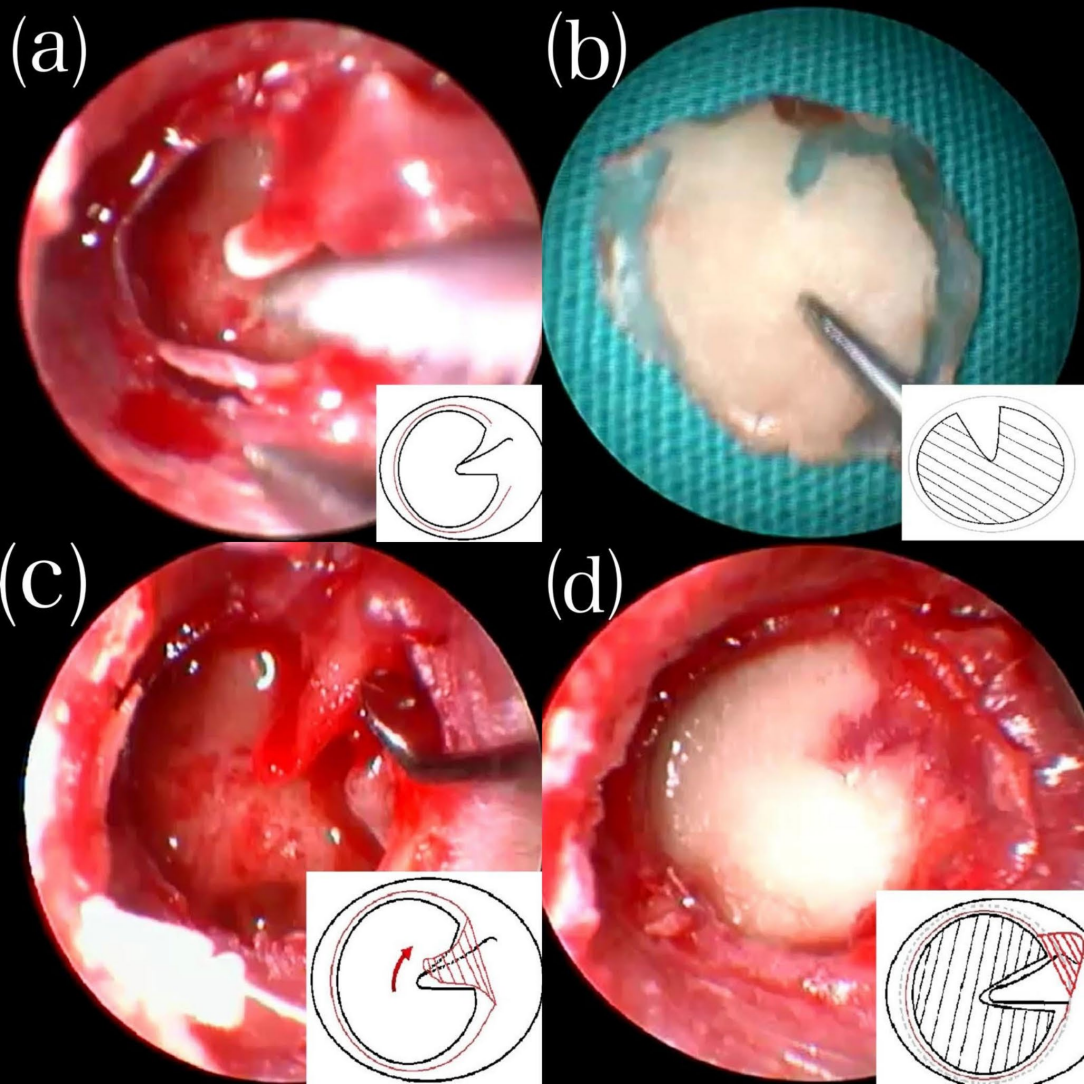
(**a**) De-epithelization and diagrammatic representation of perforation edges. (**b**) Island flap and diagrammatic representation. (**c**) The elevation and schematic representation of the membrane remnant on the lateral of the malleus. (**d**) Placement and diagrammatic representation of the island graft

**Fig. 2:**
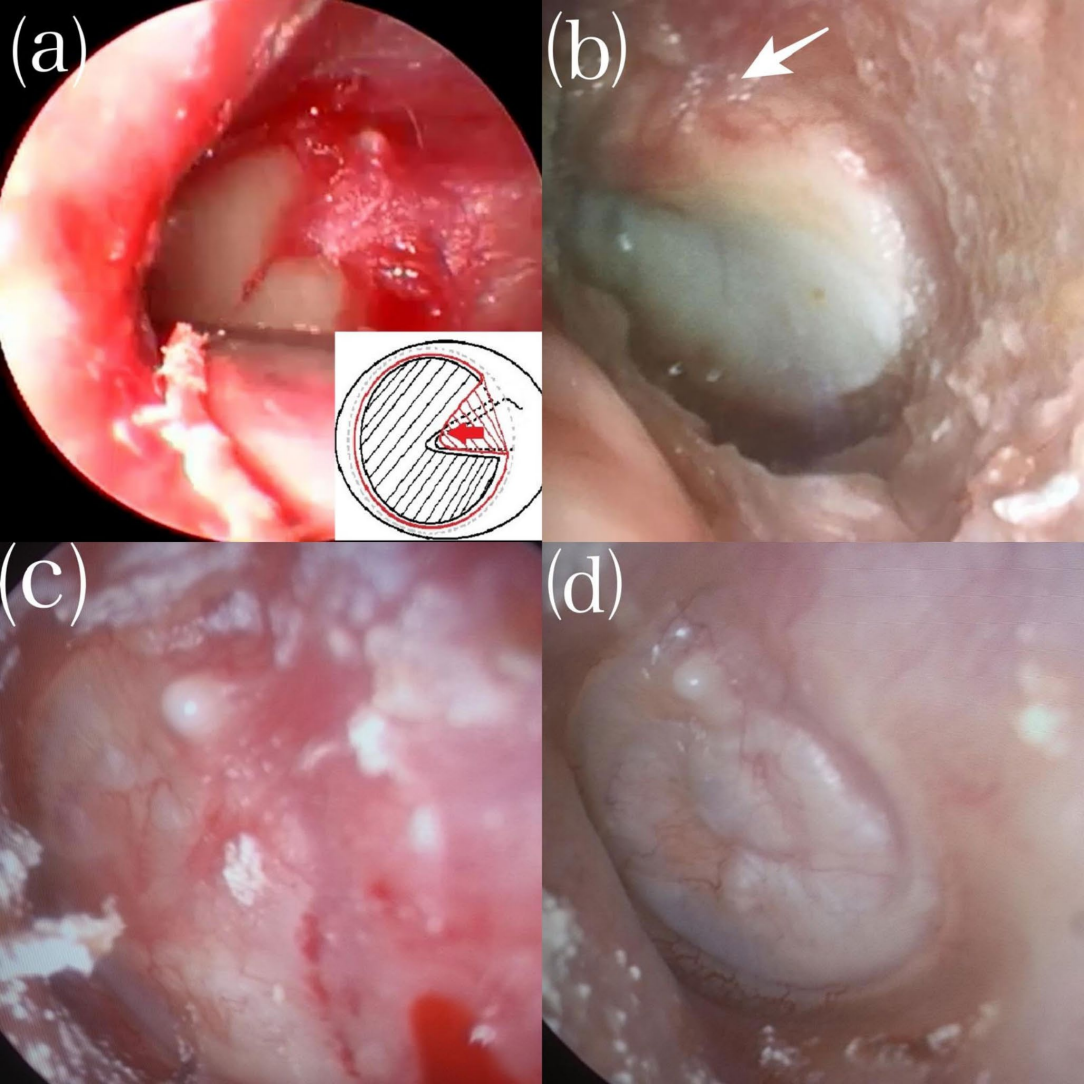
(**a**) Laying and diagrammatic representation of lateral malleus flap. (**b**) Postoperative 7th day. The arrow shows lateral malleus flap and vascularity. (**c**) Postoperative 15th day. (**d**) Postoperative second month

The surgery was started by infiltrating 0.5 cc lidocaine hydrochloride and epinephrine (Jetokain^®^) into the external auditory canal. The middle ear mucosa, bone chain movements, perforation dimensions, and any signs of chronic otitis media were carefully observed under the guidance of 0° and 30° endoscopes. Once it was confirmed that there was no pathology other than the perforation, a tragal cartilage graft was obtained. The perichondrium was preserved, and the cartilage was prepared as an island flap. The perforation edges were de-epithelized, and the membrane remnant left on the lateral side of the manubrium mallei was elevated to the neck of the malleus **(**Fig. 1a**).** The perforation dimensions were measured, and an island flap was prepared to be about 0.5 mm wider than the perforation edges, with the perichondrium on the lateral side prepared to be about 1 mm wider than the cartilage **(**Fig. 1b**).** Perforation sizes and membrane remnant left on the lateral side of the malleus were elevated as a flap to the short arm of the malleus **(**Fig. 1c**).** A groove was gently opened on the area corresponding to the malleus, and the cartilage island graft was placed gently all around the medial of the remaining membrane remnant **(**Fig. 1d**).** After ensuring the island graft was stabilized and the perforation was closed entirely, the membrane remnant elevated towards the lateral was placed back to its original position on the malleus **(**Fig. 2a**).** The surgery was completed after the graft was secured with wet sponges. The endoscopic appearance of the island graft at day 7, day 15 and month 2 were shown in Fig. 2b, c and d, respectively.

### Statistical analysis

Data analysis was performed using the statistical package for the social sciences (SPSS) for Windows, Version 22 software (SPSS Inc., Chicago, IL, USA). Continuous variables were reported as median (minimum-maximum) values. Categorical variables were stated as number (n) and percentage (%). Nominal variables were assessed by the Pearson’s chi-square or Fisher’s exact test. Mann–Whitney U test was used for comparisons of non-normally distributed parameters. Descriptive data were expressed in mean, standard deviation, median, highest and lowest values, frequency, and ratio. The Wilcoxon test was used to analyze dependent quantitative data. A *p* value of < 0.05 was considered statistically significant.

## Results

Among the 75 patients included in the study, 39 (52%) were male and 36 (48%) were female. The age of the patients ranged from 12 to 44 years, with a mean age of 26 years. A total of 70 patients (93.3%) were operated primarily, while 5 (6.7%) were operated as revision cases.

Perforation localization was subtotal in 29 patients, accounting for 38.7% of the total cohort. Central perforation was observed in 26 patients (34.7%), while posterior perforation was identified in 8 patients (10.7%). Additionally, inferior perforation occurred in 6 patients (8%), and anterior perforation was also noted in 6 patients (8%).

The perforation sizes were categorized as large in 37 patients (49.3%), medium in 24 patients (32%), and small in 14 patients (18.7%). Graft success was defined as the absence of lateralization, medialization, or perforation upon post-operative examination. The graft success rate was determined to be 94.6% (*n* = 71) among the the 75 patients included in the study.

Otoscopic examinations performed at the post-operative sixth month showed reperforation in 1 patient (1.3%), lateralization in 1 (1.3%) patient, and medialization in 2 (2.6%) patients **(**Table 1**).** The mean hearing level prior to surgery was 33.3 ± 7.0 dB, with an ABG of 24.0 ± 6.6 dB. In contrast, the post-operative mean hearing level was recorded at 18.0 ± 4.8 dB, and the ABG was 16.3 ± 5.3 dB. Statistical analysis indicated that the post-operative mean hearing level and ABG were significantly lower (*p* < 0.05) compared to the pre-operative values (Table 2).

**Table 1:** Demographic and clinical characteristics of all patients included in the study

		Min- Max	Median	Mean ± SD/ *n*-%
Age		12–44	26	26.7 ± 10.1
Gender	Male Female			39 52.0% 36 48.0%
***Hearing Level (dB)***				
500 Hz		14–46	35	32 ± 8
1000 Hz		20–45	34	31 ± 6.9
2000 Hz		20–48	34	33 ± 7.4
4000 Hz		24–48	36	35 ± 7.0
Mean Hearing Level		20–46	35	33 ± 7.0
Air Gone Gap (ABG)(dB)		10–35	25	24 ± 6.6
Duration of follow- up (months)		6–33	16	16 ± 5.3
Primary- Revision	Primary			70 93.3%
	Revision			5 6.7%
Localization	Anterior			6 8.0%
	Inferior			6 8.0%
	Posterior			8 10.7%
	Central			26 34.7%
	Subtotal			29 38.7%
Perforation Dimesion	Large			37 49.3%
	Medium			24 32.0%
	Small			14 18.7%
Reperforation				1 1.3%
Lateralization				1 1.3%
Medialization				2 2.6%

**Table 2:** Audiologic data of all patients included in the study

Hearing Level (dB)	Pre-operative		Post-operative		*p* value
	Mean ± SD	Median	Mean ± SD	Median	
500 Hz	32.2 ± 8.0	35	18.9 ± 5.3	18	***0.000*** ^w^
1000 Hz	31.8 ± 6.9	34	18.1 ± 5.1	18	***0.000*** ^w^
2000 Hz	33.4 ± 7.4	34	17.5 ± 4.9	16	***0.000*** ^w^
4000 Hz	35.9 ± 7.0	36	17.4 ± 4.8	16	***0.000*** ^w^
Mean Hearing Level (dB)	33.3 ± 7.0	35	18 ± 4.8	17.5	***0.000*** ^w^
Air Bone Gap (dB)	24 ± 6.6	25	10.9 ± 4.3	10	***0.000*** ^w^

^**w**^ **Wilcoxon test**

## Discussion

The technique used in the current study was suitable for all pars large tensa perforations. The most significant benefit of this novel technique is the reduction in surgical time, along with a decrease in postoperative pain and hospitalisation duration in comparison to alternative transcanal approaches.

In simple chronic otitis media, the repairment of the perforated membrane prevents progressive hearing loss and treats hearing loss due to membrane perforation. Generally, the procedure is called “Tympanoplasty”. Tympanoplasty is one of the most commonly performed surgical procedures by otorhinolaryngologists. Three approaches are used in the tympanoplasty procedure: endaural, transcanal, and postauricular [5]. Tympanoplasty techniques have changed over the years. Previously, large postauricular incisions were standard, but smaller endaural and transcanal incisions are now preferred. The main motivation for this change is to improve the post-operative recovery process and allow a faster return to work in the post-operative period [6].

Direct visualization of perforation edges is crucial in tympanoplasty. Traditionally, a microscope was the only tool used. The postauricular incision provides better visibility of the perforation edges than the transcanal approach in microscopic-asisted surgery. In the last thirty years, ear surgery incisions have evolved with the use of endoscopes, allowing for greater transcanal interventions, fewer incisions, and enhanced post-operative patient comfort [7].

In the conventional endoscopic approach to tympanoplasty, it is essential to elevate the tympanomeatal flap in order to provide additional support for the graft [8]. Nevertheless, the lifting of the tympanomeatal flap is associated with several disadvantages, including the potential for injury to the chorda tympani, the formation of iatrogenic colectatomas, the development of skin stenosis, the occurrence of wound infections, and an extended case duration. Schraff et al. reported the formation of epithelial pearls in two patients (22%) and the development of wound infections in one patient (11%) out of a total of nine patients [9]. In contrast, our study did not observe any of these complications.

Inlay cartilage tympanoplasty is a relatively new technique of the cartilage tympanoplasty. It was first defined by Eavey et al. in 1998, and they reported a graft success rate of 100% in all nine patients included in their study. Less morbidity was reported in the patients since the graft was obtained through an incision only in the tragus, and no postauricular or endaural incisions were made [10]. In the present study, we applied tympanoplasty without elevating the tympanomeatal flap. The aspiration requirement was lower, and the external auditory canal was less irritated during the examinations as no incisions were made to the external auditory canal, and less cerumen accumulated. Thus, patient examination in the post-operative period becomes more painless. Furthermore, no compression bandage is required. It is a more comfortable approach than the other techniques for the patient and the surgeon due to the shorter operation and post-operative recovery time.

However, an increase in the patient’s post-operative comfort is accompanied by several difficulties, including the surgeon needing to abandon the use of a microscope and perform the surgery with one hand. We observed that using the membrane remnant on the lateral side of the malleus as a superior-based flap without elevating the tympanomeatal flap shortened the duration of surgery and reduced the number of procedures that require the surgeon to use both hands during surgery. The underlay technique is the most widely accepted technique, in which the graft is placed on the medial surface of the perforated tympanic membrane.

In the tympanoplasty surgery, the prepared over-underlay graft is placed between the bone annulus and tympanomeatal flap, and tympanomeatal flap elevation helps the stabilization of the graft [11]. In the present study, we applied tympanoplasty without tympanomeatal flap elevation and used malleus to support the graft. The comparison between our tympanoplasty results and audiological records in the post-operative sixth month and tympanoplasty results reported in the current literature revealed similar findings. In the present study, the integrity of the canal was preserved by means of the over-underlay technique, and the residual membrane and malleus were employed to prevent medial mobilisation of the graft. The modified technique yielded a 94.6% success rate for grafts. Moreover, since the graft was placed over-under technique and channel integrity was preserved, the possibility of graft mobilisation decreased and its interaction with the membrane remnant was adequate. Furthermore, it is believed that the preservation of the integrity of the external auditory canal may enhance the success of grafts, as it avoids the disruption of the tissue’s blood supply. Therefore, it was thought that the migration of epithelium on the graft from the aviated tympanic membrane remnant was faster, thus achieving good graft success.

The success of the cartilage graft is high in tympanic membrane perforations. Cartilage graft is used more frequently for its durability in high-risk perforations such as eustachian tube dysfunction, revision cases, and chronic suppurative otitis media [12]. Özdamar et al. reported a cartilage graft success rate of 96%, and they did not find a significant difference in the compliance of the newly formed membrane in the high-frequency tympanometry results compared to the patients with temporal fascia grafts [13]. Onal et al. reported that the functional success of the island graft group in terms of air–bone gap closure was statistically better than temporalis fascia group [14]. In contrast to their study, Bozdemir et al. found that the air–bone gap closure was statistically better in temporalis fascia group when compared to the island cartilage group [15]. In our study, the cartilage graft success rate was 96.4%, and one patient developed lateralization while two developed medialization. The success rate for island graft retention in type 1 tympanoplasty is approximately 90%, with favourable outcomes in terms of bone-airway gap. In their study of 15 patients who had undergone island grafting and 10 patients who had undergone temporal fascia grafting, Kirazli et al. demonstrated that the success rates for the bone-airway gap in the cartilage and fascia grafts were 11.9 dB and 11.5 dB, respectively [16]. A recent meta-analysis revealed that endoscopic and microscopic butterfly cartilage graft inlay tympanoplasties demonstrate comparable anatomical and hearing outcomes. Consequently, the decision between these approaches is ultimately at the discretion of the surgeon [17]. These findings were consistent with the literature in terms of graft success.

Theoretically, an iatrogenic cholesteatoma may be a concern in the inlay technique, as in tympanoplasty without elevating the tympanomeatal flap. The follow-up duration in our study was 24 months. During this period, none of our patients developed cholesteatoma. This issue was also mentioned in the literature. In their study, Haksever et al. applied inlay cartilage tympanoplasty and reported a 96.5% graft success rate, which further improved hearing significantly. No iatrogenic cholesteatoma was developed in their study [18].

Similar to inlay cartilage tympanoplasty, the size of the perforation is a limiting factor in this technique. This study found that the technique was less effective for small perforations, possibly due to the supportive function of the malleus. Eavey and colleagues [10] observed a 100% success rate in 11 patients with minimal perforations, and noted a statistically significant improvement in hearing in 10 of these individuals. The graft success of pediatric and young patients was reported as 71% and 92%, respectively, in studies by Simon et al. [19] and Couloigner et al. [20]. In our study, the lateral malleolar flap was elevated over the malleus, unlike the inlay butterfly techniques, and this procedure was applied to patients in all age groups, in all perforations (49.3% of which were large perforations). A graft success rate of 96.4% and a statistically significant gain of 15 dB in the ABG range were achieved in our study. Similar to our study, Hod, et al. have achieved a 92% graft success rate in all age groups and all perforation dimensions and statistically significant hearing improvement in 76% of patients [21]. Our study was performed in all perforation types and all age groups, except for marginal perforations, and our graft success rate and hearing improvement were compatible with the literature.

Although thinner graft materials were hypothesized to improve membrane vibration and sound transmission, existing literature suggests that they do not detrimentally affect post-operative hearing outcomes, regardless of the cartilage thickness used for perforation closure or the perforation size [22–24]. Despite the thickness and stiffness of the cartilage, our hearing results are compatible with the literature.

The limitation of our study is the marginal perforations not being included. In this type of perforation, the graft can be placed medial to this skin with minimal elevation of the external auditory canal skin that forms the perforation edge. There is a technical risk of developing cholesteatoma in the membrane epithelium under the enlarged perichondrium in our technique. Therefore, it is essential to remove this epithelium. The follow-up period of our patients was approximately 24 months, and no patient developed cholesteatoma. Another limitation of the study is the low number of patients collected, although the present study represents the largest currently published due to its recent introduction. Further studies involving a larger sample of patients and a longer follow-up period are needed to confirm the data presented in this study.

## Conclusion

The results of endoscopic tympanoplasty performed with lateral malleolar flap without elevating the external auditory canal in the present study are similar to the results of other studies reported in the literature using other techniques. This method may be an alternative option for surgeons applying endoscopic tympanoplasty.

## Data Availability

Data are available upon reasonable request from the corresponding author.
